# Supplementary material for: Tocilizumab Evaluation in HLA-Desensitization before Kidney Transplantation as an Add-On Therapy to Apheresis: The TETRA Study
Source: J Clin Med. 2023 Jan 4;12(2):424. doi: 10.3390/jcm12020424 (PMC9866000; doi:10.3390/jcm12020424)
Supplement: Supplementary file 1 [file jcm-12-00424-s001.zip › jcm-2080798-supplementary.docx]

**Supplementary Figure S1.** Kidney function trajectories, among the two groups.
